# Comparison of Brock University, Mayo Clinic and Herder models for pretest probability of cancer in solid pulmonary nodules

**DOI:** 10.1111/crj.13546

**Published:** 2022-10-07

**Authors:** Seher Susam, Akın Çinkooğlu, Kenan Can Ceylan, Soner Gürsoy, Berna Eren Kömürcüoğlu, Aydan Mertoğlu, Ali Kadri Çırak, Mine Gayaf, Filiz Güldaval, Fevziye Tuksavul, Gülru Polat, Sena Ataman, Eylem Yıldırım, Hakan Koparal, Nur Yücel

**Affiliations:** ^1^ Department of Radiology, Dr. Suat Seren Chest Disease and Thoracic Surgery Training and Research Hospital Health Sciences University Izmir Turkey; ^2^ Department of Thoracic Surgery, Dr. Suat Seren Chest Disease and Thoracic Surgery Training and Research Hospital Health Sciences University Izmir Turkey; ^3^ Department of Chest Disease, Dr. Suat Seren Chest Disease and Thoracic Surgery Training and Research Hospital Health Sciences University Izmir Turkey; ^4^ Department of Nuclear Medicine, Dr. Suat Seren Chest Disease and Thoracic Surgery Training and Research Hospital Health Sciences University Izmir Turkey; ^5^ Department of Pathology, Dr. Suat Seren Chest Disease and Thoracic Surgery Training and Research Hospital Health Sciences University Izmir Turkey

**Keywords:** Brock University, Herder, Mayo Clinic, pulmonary nodule, risk

## Abstract

**Objective:**

Risk analysis models, which are used in the diagnostic algorithm of incidental pulmonary nodules, are based on patient data from developed countries. Mayo Clinic, Brock University and Herder are among the most known models. We aim to compare the reliability of these models in patients with indeterminate solid nodules and to investigate the contribution of the predictors used to the model.

**Methods:**

We analysed 305 patients who performed transthoracic needle biopsy and positron emission tomography/computed tomography for solid nodules, retrospectively. For all three models, the malignancy risk probabilities of patients were calculated, and patients were classified as low (<5%), moderate (60%) and high (<60%) risk groups. Later, the malignancy rates of each model in three different risk groups were compared within each other and among the models.

**Results:**

The malignancy rate is 73% in 305 patients. In the Mayo Clinic and Herder models, the difference in the low‐, medium‐ and high‐risk groups is significant (*p* < 0.001). In the medium‐risk group, the rate of malignancy is 96.8% in the Brock model. In the high‐risk group, the rate of malignancy in Herder is 88.3% and the rate of malignancy in Mayo Clinic is 28.8%. The optimal cutoff values for the Mayo Clinic, Brock University, and Herder were 29.6, 13.4 and 70 (AUC, *respectively*; 0.71, 0.67 and 0.73). Age, smoking, gender, size, emphysema and spiculation increase the likelihood of malignancy.

**Conclusion:**

Close results were obtained in all three models. In the high‐risk group, the Herder model has the highest reliability rate (odds ratio 3.3, confidence interval [1.1, 10.2]). Upper lobe predilection is not a reliable predictor.

## Graphical abstract

We evaluate Mayo Clinic, Brock University and Herder prediction models to estimate the pretest probability of malignancy in solid pulmonary nodules. In 305 patients with a high malignancy rate (73%) who underwent PET/CT and transthoracic needle biopsy, the results were close in all three models. However, the Herder model was found to be more successful in the high‐risk group (60%).

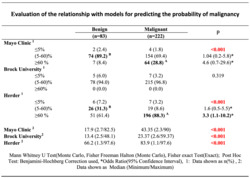

## 1 INTRODUCTION

The most crucial problem in pulmonary nodules detected incidentally on computed tomography (CT) can quickly, reliably and economically distinguish benign and malignant nodules.^1^, ^2^, ^3^ Because CT has no diagnostic morphological findings for malignancy, an additional examination is required.^4^, ^5^ The selection of these tests is clinical prediction models that predict the probability of the patient's malignancy. Models developed by Mayo Clinic, Brock University and Herder are some of them.^6^, ^7^, ^8^ Almost all these models are based on the data of developed countries.^9^, ^10^, ^11^ However, it is a fact that cancer and infection rates, smoking rate, environmental risk factors and average life expectancy are different in underdeveloped countries.^12^, ^13^ Our aim is to compare the reliability of these three models in the differential diagnosis of benign and malignant nodules and to analyse the risk of malignancy of the parameters based on the data of our country.

## 2 MATERIAL AND METHODS

### 2.1 Patients

This study is approved by the Medical Practice Training Committee of our hospital. All patients who went through transthoracic needle biopsy (TTNB) and positron emission tomography (PET) between 2012 and 2017 in our hospital were retrospectively examined. Among these, patients with a solid nodule less than 3 cm were detected. Intervals of more than 1 month between positron emission tomography/computed tomography (PET/CT) and TTNB, cases with consolidation, pleural fluid, mediastinal lymphadenopathy and patients without a definitive diagnosis were excluded from the study. We also took cases with known primary malignancy and multiple nodules. Patients who were diagnosed histopathologically with surgical biopsy were considered as a definitive diagnosis. Nodules whose cytology were benign, whether they reduced in size, disappeared or remained stable at 2 years of follow‐up, were accepted benign. Patients with cytology malignant but inoperable were considered malignant if they had radiological progression or another focus was detected. Information about risk factors such as smoking, cancer history in the family and in itself was obtained from the data recorded in the system.

### 2.2 CT imaging

In our hospital, a multidetector‐row CT scanner with maximum section collimation of 1.25–2.5 mm and four or more data‐acquisition channels was used. Two radiologists who had specific training in chest radiology reviewed the CT scans Images were analysed in the lung parenchyma and mediastinum window. First of all, nodules with partial solid and ground glass appearance were eliminated. Solid nodule size was accepted as the average of the longest and shortest diameter perpendicular to each other in the parenchyma window in the axial plane. Spiculation in the nodule contour, the presence of emphysema, localization of the upper lobe and the number of nodules were recorded.

### 2.3 PET/CT imaging

PET/CT images were obtained by PHILIPS Gemini TF 16 Slice PET/CT scanner. Patients with a blood glucose level <200 mg/dl after at least 6 h of fasting were given 7–9 MCI IV F‐18 FDG. One hour after injection, images of the patients from the vertex to the thigh were captured. CT (140 kV, 100 mAs, 5‐mm slice thickness) and PET/CT (1.5 min/bed position) images were obtained sequentially and combined in workstations. CT data were used for attenuation correction. An isocontour 3D region of interest (ROI) was plotted to contain the lung parenchymal lesion. The maximum pixel count in the ROI was determined as the maximum standardized uptake value (SUV max). According to the visual categorization in Herder's original article and the general practice in our hospital, four groups were determined quantitatively. We classified that: If SUV max value is <1: “no uptake”; 1 < SUV max < 1.5: “faint uptake”; 1.5 < SUV max < 2.5: “moderate uptake”; 2.5 < SUV max: “intense uptake.”^13^, ^14^, ^15^

### 2.4 Risk analysis and statistics

According to the Mayo Clinic, Brock University and Herder models, a quantitative evaluation was done on the web with all the data of the patients. Patient age, nodule size, smoking history, cancer without a 5‐year limit, upper lobe involvement and spiculation parameters were used for the Mayo Clinic model.^10^ In the Brock University model, gender, age, nodule size, family history of cancer, emphysema, number of nodules, solid nodule, upper lobe involvement and spiculation parameters were entered.^9^ In Herder model, age, cigarette history, cancer history, size, spiculated edge, upper lobe involvement and FDG uptake data were entered.^11^

The risk probability for each model was classified as low if <5%, moderate if 5%–60% and high if <60%. In this distinction of risk groups, the 2007 American College of Chest Physician (ACCP) guideline was referenced.^1^

The analysis of the variables was performed via SPSS 25.0 (IBM Corp. New York) program. Below each table, the methods of the measured parameters are detailed. Variables were examined at the 95% confidence interval (CI).

## 3 RESULTS

In 305 patients with a mean age of 61.9 ± 9.8 (27–84), 27% benign (83/305) and 73% (222/305) malignant nodules were detected. Demographic characteristics of patients and analysis of the factors associated with malignancy can be seen in Table 1, with Benjamin Hochberg correction. In univariate analysis, there was no significant relationship between the presence of emphysema, the upper lobe localization of the lesion, the history of cancer in the family, in the person, and its malignancy (*p* > 0.05). Malignancy rate was statistically more significant in males (odds ratio [OR] 2.02, 95% CI [1.13, 3.62], *p* = 0.019), smokers (OR 3.04, 95% Cl [1.7, 5.3], *p* < 0.001), spicular contoured nodules (OR 1.97, 95% Cl [1.18, 3.31], *p* = 0.01). Age, size and SUV max values of malignant patients were significantly higher than those of benign (*p* < 0.001, <0.001 and <0.001, respectively). The number of nodules was higher in those who were benign (*p* = 0.043).

**TABLE 1:** Demographic characteristics of patients and evaluation of risk factors associated with malignancy

		Benign	Malignant	*P*
		(*n* = 83)	(*n* = 222)	
Gender^a^				
	Female	25 (30.1)	39 (17.6)	0.019
	Male	58 (69.9)	183 (82.4)	2.02 (1.13–3.62)^c^
Age^b^		57 (33/77)	63 (27/84)	<0.001
Nodule number^b^		1.5 (1/10)	1 (1/16)	0.043
Size^b^		14.5 (8.5/20)	16.15 (10.1/20)	<0.001
SUV max^b^		3.55 (1/19.6)	7.15 (0.5/36)	<0.001
Cigarette^a^				
	No	32 (38.6)	38 (17.1)	<0.001
	Yes	51 (61.4)	184 (82.9)	3,04 (1.7–5.3)^c^
Emphysema^a^				
	No	42 (50.6)	91 (41.0)	0.154
	Yes	41 (49.4)	131 (59.0)	
Upper lobe^a^				
	No	32 (38.6)	90 (40.5)	0.794
	Yes	51 (61.4)	132 (59.5)	
Spiculation^a^				
	No	52 (62.7)	102 (45.9)	0.01
	Yes	31 (37.3)	120 (54.1)	1.97 (1.18−/3.31)^c^
Cancer history in the family^a^				
	No	76 (91.6)	191 (86.0)	0.244
	Yes	7 (8.4)	31 (14.0)	
Primary CA^a^				
	No	73 (88.0)	177 (79.7)	0.131
	Yes	10 (12.0)	45 (20.3)	

*Note*: Mann Whitney *U* test (Monte Carlo), Pearson *χ* ^2^ test (Exact).

^a^ Data shown as *n* (%).

^b^ Data shown as median (minimum/maximum).

^c^ Odss Ratio (95% confidence interval).

The median values of malignant nodules were higher in all three models than in the benign groups (*p* < 0.001). When comparing malignancy rates of low‐, medium‐ and high‐risk groups for each model, the Brock was statistically insignificant (*p* = 0.319). For benign nodules in the medium‐risk group in the Mayo Clinic model, the OR is 1.04 (95% CI [0.2, 5.8]), in the Herder model, OR is 1.6 (95% CI [0.5, 5.5]) but was not statistically significant. In the Mayo Clinic, the malignancy rate in those with ≥60% risk is higher than those with ≤5% (OR 4.6, 95% CI [0.7, 296], *p* > 0.001). The result is similar in the Herder model (OR 95% CI [1.1, 10.2]) (Table 2).

**TABLE 2:** Evaluation of the relationship with models for predicting the probability of malignancy

		Benign	Malignant	*p*
		(*n* = 83)	(*n* = 222)	
Mayo Clinic^a^				
	≤5%	2 (2.4)	4 (1.8)	<0.001
	5%–60%	74 (89.2)^B^	154 (69.4)	1.04 (0.2–5.8)^c^
	≥60%	7 (8.4)	64 (28.8)^A^	4.6 (0.7–29.6)^c^
Brock University^a^				
	≤5%	5 (6.0)	7 (3.2)	0.319
	5%–60%	78 (94.0)	215 (96.8)	
	≥60%	0 (0.0)	0 (0.0)	
Herder^a^				
	≤5%	6 (7.2)	7 (3.2)	<0.001
	5%–60%	26 (31.3)^B^	19 (8.6)	1.6 (0.5–5.5)^c^
	≥60%	51 (61.4)	196 (88.3)^A^	3.3 (1.1–10.2)^c^
Mayo Clinic^b^		17.9 (2.7/82.5)	43.35 (2.3/90)	<0.001
Brock University^b^		13.4 (2.5/48.1)	23.37 (2.6/59.37)	<0.001
Herder^b^		66.2 (1.3/97.6)	83.9 (1.1/97.6)	<0.001

*Notes*: Mann Whitney *U* test (Monte Carlo, Fisher Freeman Halton, Monte Carlo), Fisher exact test (Exact); post hoc test: Benjamini–Hochberg correction used.

^a^ Data shown as *n* (%).

^b^ Data shown as median (minimum/maximum).

^c^ Odds ratio (95% confidence interval).

For the Mayo Clinic, Brock and Herder models, when the benign and malignant ratios of the data in the low‐, medium‐ and high‐risk groups were compared, no statistical significance was found between the three models for benign lesions in the low‐risk group (*p* = 0.167, 0.27, 0.672, respectively). In the medium‐risk group, the Herder model had a lower benign nodule rate compared with the others (*p* = 0.066). In the high‐risk group, while the benign nodule rate in Mayo Clinic was significantly lower than Herder, no comparison between Brock and Mayo Clinic and Herder was made (*p* < 0.001). For malignant nodules, when the rates of Mayo Clinic and Brock, Mayo Clinic and Herder, and Herder and Brock were compared, no significance was found (*p* = 0.236, 0.236, 0.999, respectively). In the 5%–60% risk group, the malignant incidence in Brock was higher (*p* < 0.001) than the others, and Mayo Clinic had a higher malignancy rate than Herder (*p* < 0.001). While the malignancy rate in Herder in the high‐risk group was significantly higher than in Mayo Clinic, no comparison with Brock and Mayo Clinic and Herder was made (*P* < 0.001) (Table 3).

**TABLE 3:** Comparison of benign and malignant nodule ratios of Mayo Clinic, Brock and Herder models according to the probability of risk

			Benign	Malignant
			(*n* = 83)	(*n* = 222)
			*n* (%)	*n* (%)
Mayo Clinic				
		≤5%	2 (2.4)	4 (1.8)
		5%–60%	74 (89.2)	154 (69.4)
		≥60%	7 (8.4)	64 (28.8)
Brock University				
		≤5%	5 (6.0)	7 (3.2)
		5%–60%	78 (94.0)	215 (96.8)
		≥60%	0 (0.0)	0 (0.0)
Herder				
		≤5%	6 (7.2)	7 (3.2)
		5%–60%	26 (31.3)	19 (8.6)
		≥60%	51 (61.4)	196 (88.3)
*p* value for pairwise proportion comparison	*p* (Mayo Clinic vs. Brock)			
		≤5%	0.167	0.236
		5%–60%	0.066	<0.001
		≥60%	—	—
	*p* (Mayo Clinic vs. Herder)			
		≤5%	0.279	0.236
		5%–60%	<0.001	<0.001
		≥60%	<0.001	<0.001
	*p* (Herder vs. Brock University)			
		≤5%	0.672	0.999
		5%–60%	<0.001	<0.001
		≥60%	—	—

*Note*: One proportion Pearson *χ* ^2^ test (Exact), According to Bonferroni correction, if *p* < 0.0167, it will be considered significant.

When our variables related to malignancy are included in the model; gender (OR = 2.209, 95% Cl [0.930, 5.251]), age (OR = 1.097, 95% CI [1.058, 1.137]), size (OR 1.219, 95% CI [1.077, 1.379]), SUV max (OR 1.176, 95% CI [1.081, 1.279]), smoking history (OR 2.439, 95% CI [1.021, 5.83]) and emphysema (OR 2.498 95% CI [1.117, 5.586]) increase malignancy. This model accurately estimates 93.2% of malignant nodules, 47.6% of benign and 80.9% of all cases in general (*P* < 0.001) (Table 4).

**TABLE 4:** Evaluation of the factors associated with malignancy by multivariate analysis

	*B*	S.H.	*p*	Odds ratio	Odds ratio (95% CI)	
					Lower limit	Upper limit
Gender	−0.793	0.442	0.073	2.209	0.930	5.251
Age	0.092	0.019	<0.001	1.097	1.058	1.137
Nodule number	−0.110	0.086	0.204	1.116	0.942	1.322
Size	0.198	0.063	0.002	1.219	1.077	1.379
SUV max	0.162	0.043	<0.001	1.176	1.081	1.279
Cigarette	−0.892	0.445	0.045	2.439	1.021	5.830
Emphysema	0.915	0.411	0.026	2.498	1.117	5.586
Upper lobe	−0.022	0.336	0.947	1.022	0.530	1.974
Spiculation	−0.180	0.378	0.634	1.197	0.570	2.513
Cancer history in the family^a^	−0.581	0.565	0.304	1.788	0.591	5.411
Primary another cancer	−0.580	0.481	0.228	1.787	0.696	4.591
Constant	−6.215	1.607	<0.001			

*Notes*: The dependent variable: malignancy predicted (malignant) = 93.2; predicted (benign) = 47.6; predicted (overall) = 80.9; *p* model<0.001. Multiple logistic regression (method = enter), G.A, confidence interval—B: regression coefficients—SH, standard error.

The performance of separation of the risk analysis models for benign and malign cases according to the optimal cut off value is evaluated. In the Mayo Clinic model, the sensitivity, specificity and overall accuracy results for the 29.6 cut‐off value are 68.9%, 67.5% and 68.5%, respectively (*area under the ROC curve* [AUC] 0.711). In the Brock model, for 13.4 cut‐off value, 75.2%, 51.8%, and 68.8% (AUC 0.667), in the Herder model, for 70 cutting value, 74.3%, 63.9% and 71.5%, and (AUC 0.727). Table 5 shows the *p*‐value (*p* < 0.001) obtained from the ROC curve analysis performed for the success of every three model's benign and malignant differentiation. AUC in all the models are 0.727, 0.711 and 0.667, respectively. When model performances are compared with each other, there was no significant difference between AUC values between all three models (*p* = 0.351, 0.732, 0.205) (Table 5).

**TABLE 5:** Evaluation of separation performance of benign and malignant cases of risk analysis models according to optimal cut‐off value

		Benign	Malignant	Accuracy rate (%)	AUC (Se)	*p*	*p* value for comparison AUC
		*n* (%)	*n* (%)				
Mayo Clinic							
	≤29.6	56 (67.5)^sp^	69 (31.1)	68.5	0.711 (0.033)	<0.001	0.351 ^Mayo cln‐‐Brock^
	>29.6	27 (32.5)	153 (68.9) ^ss^				
Brock University							
	≤13.4	43 (51.8) ^sp^	55 (24.8)	68.8	0.667 (0.034)	<0.001	0.732 ^Mayo cln ‐‐Herder^
	>13.4	40 (48.2)	167 (75.2) ^ss^				
Herder							
	≤70	53 (63.9) ^sp^	57 (25.7)	71.5	0.727 (0.033)	<0.001	0.205 ^Brock‐‐Herder^
	>70	30 (36.1)	165 (74.3) ^ss^				
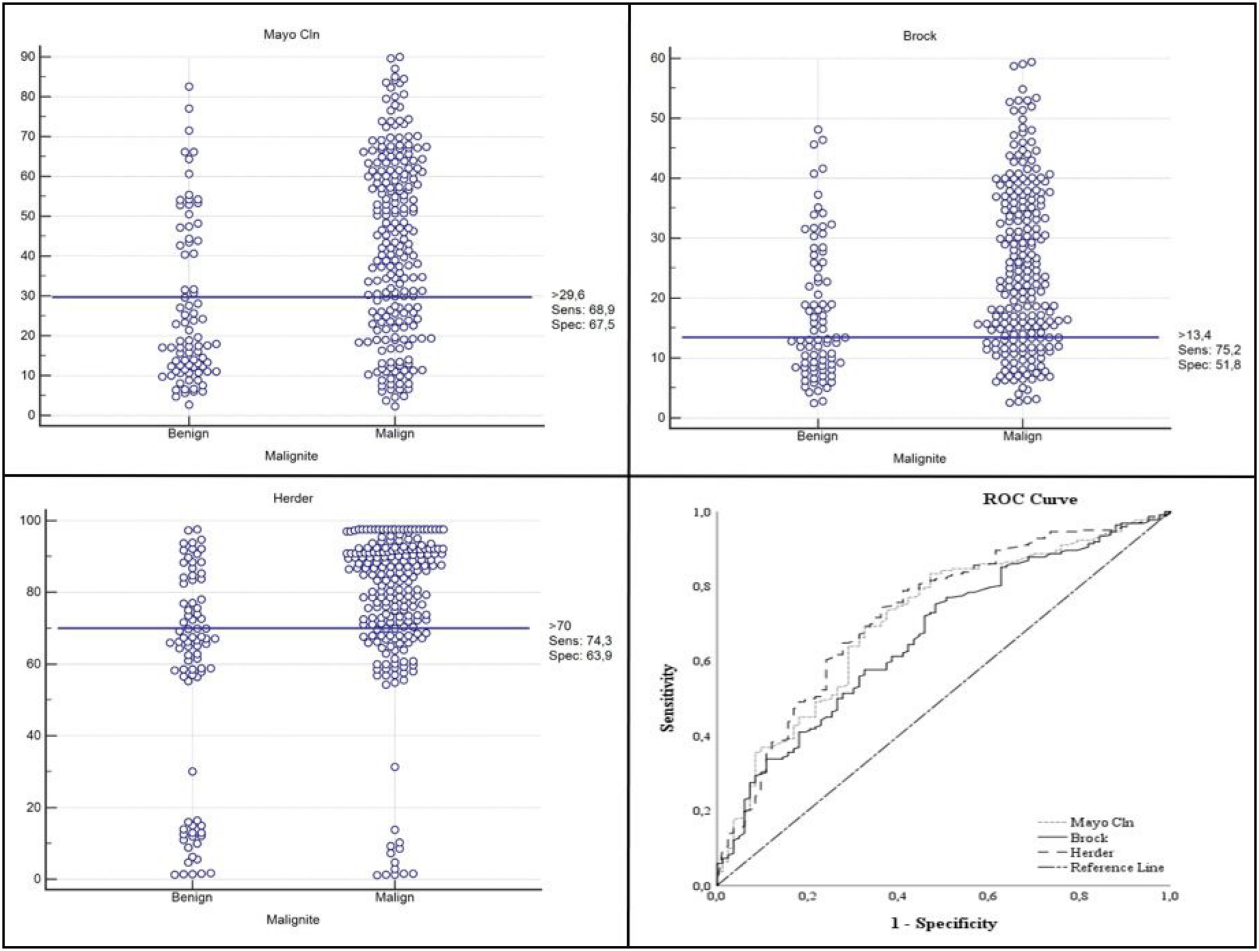							

Abbreviations: AUC, area under the ROC curve; ROC, receiver operating curve analysis (Honley&Mc Nell—Youden index J); Se: standard error; sp: specificity; ss, sensitivity.

## 4 DISCUSSION

Incidental pulmonary nodules appear with an increasing frequency every day, as well as bring preference confusion among alternative diagnostic methods. Institutions having a voice in pulmonary nodule management such as Fleishner Society (FS), ACCP and British Thoracic Society (BTS) recommend malignancy risk analysis before proceeding to the next stage in the diagnostic algorithm.^3^, ^6^, ^11^, ^12^, ^13^, ^14^, ^15^, ^16^, ^17^ According to the results of the risk analysis, follow‐up in the low‐risk group, PET/CT +/− TTNB, and surgery in the high‐risk group are generally recommended.^5^, ^8^, ^18^ However, today the desired level has not been reached in many countries regarding the selection and application of risk analysis methods. Studies among pulmonologists show that the rates of biopsy and surgery performed in patients in the low‐, medium‐ and high‐risk groups are approximately similar.^8^ First of all, the clinician should believe in the necessity of risk analysis, choose the most appropriate method considering the patient population and be able to trust the test result. The most widely used and well‐known risk analysis methods such as Mayo Clinic, Brock University, Veterian analysis and Herder are based on patient data obtained from developed countries. The biggest problem in pulmonary nodule management between countries with high socioeconomic status and those with low socioeconomic status is distinguishing cancer from infection. Otherwise, the rate of unnecessary interventions or operations will be extremely high. The aim of this study is also to compare the application results of these methods in our country, where infections or infestations such as tuberculosis and hydatid cysts are common, with the results of studies conducted in other countries.

In this study, persistent subsolid nodules were excluded because of their higher probability of malignancy and different management, and the probability of predicting benign and malignant nodules of the Mayo Clinic, Brock University and Herder methods were compared in solid nodules. There are many external validation studies that compare prediction models for each model separately or in pairs.

In all three models, the risk of malignancy was higher in malignant nodules. Higher reliability in the Herder model is compatible with the literatüre.^2^, ^3^, ^7^, ^19^ The reason for the absence of a significant difference in malignancy rates in the low‐risk group is that our cases required biopsy and PET/CT, and were in the patient profile with a high pretest suspicion rate and the insufficient number of patients for reliable statistics in the low‐risk group. In our study, there are not only solid nodules that are followed. The rate of malignancy is also high in Herder (55%) who chose patients with PET/CT and treat (72%) working with patients undergoing surgery.^3^, ^7^, ^15^, ^20^

The differences between the models become evident in the medium‐risk group (5%–60%). The Brock model estimated the probability of malignancy in those with a moderate risk higher than the Mayo Clinic. The Mayo Clinic model developed by Swensen was developed with nodules detected on the X‐ray.^3^, ^10^ It is a significant limitation not to apply it in patients with malignancy and a history of lung cancer in the past 5 years, because in daily practice, there are many such patients. 19% of our patients have a primary malignancy in the last 5 years. In these patients, it is observed in studies that the probability of nonmetastatic nodules is not low.^2^ The OR we obtained for the cancer history in the person is 1.787 and for the Mayo Clinic is 1.3388 in patients with cancer before 5 years. Our rate of metastasis is high as we have taken patients with a history of cancer in the last 5 years. It was observed that the upper lobe localization was not effective in the benign–malignant distinction. It is thought that infections such as tuberculosis are more common in our country, and these infections tend to settle in the upper lobes. Mayo Clinic in 1997, Herder in 2005 and Brock in 2013 made evaluations with logistic regression analysis. In our research, we preferred this method rather than Bayesian analysis too.^3^ According to the literature, 0.67 AUC is low. Another problem for those who apply this model is to leave the risk group cut‐off values to the clinician.^9^ In our study, we used the risk rates of <5%, 5%–60% and <60% recommended by ACCP. The higher the threshold, the higher the specificity and the lower the sensitivity. Reliability is 68.8% for the optimal cut off (>29.6) we have achieved.

In the Brock model, we could not evaluate the family history of cancer due to insufficient data recorded. It is necessary to know very well which cancers to include, how many generations to go, whether old or new. Genetic predisposition does not apply to all cancer groups. This modelled nodule attenuation is also desired, but the absence of partial solid and ground‐glass nodules is one reason that no patients are seen in the high‐risk group in this model. Emphysema is a risk factor for malignancy in accordance with the literatüre.^10^ As the number of nodules increases, the probability of being benign increases. The Brock model provides a better estimation than the Mayo Clinic only in terms of age, gender, size, and spiculation risk factors. Annette Williams Brock University developed the risk model for nodules with a cancer incidence of 5.5%, detected in screening studies, and most of them are smaller than 1 cm.^9^ It is thought that hospital‐based, high cancer rate, and X‐ray‐based retrospective research results are not suitable for screening studies.^8^ However, BTS recommends the Brock model for all nodules detected in incidental or screening studies, as in our patient group.^16^

According to BTS, risk groups for the Brock model have 10% and 70% cut‐off values.^16^ In our data, a 13.4% cut‐off was obtained for the Brock model. According to this value, our reliability is statistically at 68.8%. However, prospective studies are needed in which the data are complete, including nodules that are solid, subsolid, and smaller than 1 cm.

Herder added functional evaluation to the Mayo Clinic model with FDG uptake.^14^ Consistent with the literature, the AUC increase is about 0.13, but there is a slight increase in our evaluation.^3^, ^14^ PET/CT is recommended as a diagnostic method before TTNB in nodules larger than 8 mm in algorithms, especially in low‐risk patients.^14^, ^18^, ^21^, ^22^ Despite its high sensitivity and low specificity, it is more effective in preventing unnecessary invasive procedures. At this point, the most crucial problem is the SUV max cut‐off value. In Herder's original work, a qualitative evaluation was made as ‘absent, faint, moderate, intense’.^14^, ^15^, ^23^, ^24^ We accepted the 2.5 SUV max we used in our daily practice to cut‐off. Cut‐off value goes up to 8 SUV max in some studies.^25^, ^26^

Size, contour, localization, age and smoking history are clinical and radiological risk factors in both the Mayo Clinic and Brock models, and their relationship with the original cohort studies has been shown.^9^, ^10^ It is compatible with our results except for the upper lobe localization. Due to the prevalence of infections such as tuberculosis in our country, the upper lobe parameter in all three models was not statistically significant in our results. There is a similar approach to this parameter in the Asia group.^12^ Known primary malignancy is a vital risk parameter; the opinion that patients with a history of cancer in the last 5 years should be taken directly to PET/CT and biopsy in daily practice. It is thought that a history of cancer should be mentioned before 5 years.

The AUC represents the probability that the model correctly determines if a PN is malignant or benign. Because it shows the highest sensitivity and specificity, evaluations were made on this value in the literature. In the external validation study of Nair et al. conducted in 2018 for nodules larger than 8 mm in a group of fully smokers, the malignancy rate was 8.8%, AUC 0.82 in the Mayo Clinic model, and 84% in Brock. According to the results of Soardi's study in 200 patients with a malignancy rate of 54.5% in 2017, the AUC for the Mayo clinical model is 0.60. In 2017, Perandini reported 0.92 AUC in the Herder model in 259 patients with a 59% malignancy rate. In the validation study of Sher et al. in a group of 1798 patients with a 66.6% malignancy rate, the AUC for Mayo Clinic was 0.80, and 0.83 for Brock In 2015, Al Ameri reported AUC values as 0.89 for Mayo Clinic, 0.92 for Herder and 0.90 for Brock in 244 patients with a 40.6% prevalence of malignancy.^7^, ^19^, ^27^, ^28^, ^29^ According to our results, AUC is 0.71 for Mayo Clinic, 0.67 for Brock and 0.73 for Herder.

The Mayo clinical model is recommended in low‐ or malignant‐risk populations, and in high‐risk groups, as in our result, success is lower.^3^ The reliability of the Herder model is higher in comparative studies than the Mayo Clinic.,^3^, ^7^ ^30^ Brock is recommended for cancer screening studies, but it is also successful in high‐risk groups.^7^, ^27^ The difference is that it takes into account nodule multiplicity and is the only method that distinguishes solid and subsolid nodules, but our study is only for solid nodules. Therefore, the Herder model is considered more appropriate for patients in the high‐risk group with a high rate of malignancy, both in societies with high socioeconomic status and in countries like ours where infections such as tbc are relatively common.

The limitations of this study are the high risk of malignancy and the group of patients requiring biopsy and PET/CT. Our benign nodule rate is low. The fact that the study is retrospective and some missing information in the hospital records are also a significant limitation. Patients with cancer in the last 5 years were also included in the study. The absence of subsolid and nodules smaller than 1 cm also restricts the use of the results for all nodules. In order to develop new risk models suitable for the conditions of our country, prospective studies should be planned with the addition of multicentre, more patients, and new predictors.

## 5 CONCLUSION

Herder model was found to be more successful in predicting the possibility of malignancy in patients with solid pulmonary nodules than the Mayo Clinic and Brock University. Close results were obtained in all three models. It is seen that age, gender, smoking history, spiculation, size and FDG involvement are reliable predictors, and more reliable results are obtained with classification as a low‐ and medium‐high group.

## AUTHOR CONTRIBUTIONS

Seher Susam: conceptualization (lead), writing of the original draft (lead), investigation (lead), methodology (equal). Akın Çinkooğlu: resources (lead) and validation (equal). Kenan Can Ceylan: data curation (lead). Soner Gürsoy: methodology (lead). Berna Eren Kömürcüoğlu: project administration (lead). Aydan Merdoğlu: supervision (equal). Ali Kadri Çırak: supervision (equal). Mine Gayaf: formal analysis (lead), resources (equal). Filiz Güldaval: validation (equal). Fevziye Tuksavul: supervision (equal). Gülru Polat: writing review and editing (lead). Sena Ataman: investigation (equal). Eylem Yıldırım: validation (lead): Hakan Koparal: visualization (equal). Nur Yücel: methodology (equal).

## CONFLICT OF INTEREST

The authors declare that they have no conflict of interest.

## ETHICS STATEMENT

Date: 20/06/2018, Number: 49109414‐806.02.02.

## Data Availability

The data that support the findings of this study are openly available in NODÜL 3 risk grubu at https://drive.google.com/drive/u/0/my-drive.
